# High fat diet enhances cardiac abnormalities in SHR rats: Protective role of heme oxygenase-adiponectin axis

**DOI:** 10.1186/1758-5996-3-37

**Published:** 2011-12-23

**Authors:** Jian Cao, Komal Sodhi, Nitin Puri, Sumit R Monu, Rita Rezzani, Nader G Abraham

**Affiliations:** 1 Department of Geriatric Cardiology, Chinese PLA General Hospital, Beijing 100853, China; 2 Department of Physiology and Pharmacology, College of Medicine, University of Toledo, Toledo, Ohio, 43614, USA; 3 Department of Biomedical Science, Division of Anatomy, University of Brescia, Brescia Italy

**Keywords:** heme oxygenase, adiponectin, high fat diet, COX-2, oxidative stress

## Abstract

**Background:**

High dietary fat intake is a major risk factor for development of cardiovascular and metabolic dysfunction including obesity, cardiomyopathy and hypertension.

**Methods:**

The present study was designed to examine effect of high fat (HF) diet on cardio-vascular structure and function in spontaneously hypertensive rats (SHR), fed HF diet for 15 weeks, a phenotype designed to mimic metabolic syndrome.

**Results:**

Development of metabolic syndrome like phenotype was confirmed using parameters, including body weight, total cholesterol and blood pressure levels. High fat diet impaired vascular relaxation by acetylcholine and exacerbated cardiac dysfunction in SHRs as evidenced by lower left ventricular function, and higher coronary resistance (CR) as compared to controls (p < 0.05). The histological examination revealed significant myocardial and peri-vascular fibrosis in hearts from SHRs on HF diet. This cardiac dysfunction was associated with increased levels of inflammatory cytokines, COX-2, NOX-2, TxB2 expression and increase in superoxide (O_2_^-^) levels in SHR fed a HF diet (p < 0.05). HO-1 induction via cobalt-protoporphyrin (CoPP,3 mg/kg), in HF fed rats, not only improved cardiac performance parameters, but also prevented myocardial and perivascular fibrosis. These effects of CoPP were accompanied by enhanced levels of cardiac adiponectin levels, pAMPK, peNOS and iNOS expression; otherwise significantly attenuated (p < 0.05) in HF fed SHRs. Prevention of such beneficial effects of CoPP by the concurrent administration of the HO inhibitor stannic mesoporphyrin (SnMP) corroborates the role of HO system in mediating such effects.

**Conclusion:**

In conclusion, this novel study demonstrates that up-regulation of HO-1 improves cardiac and vascular dysfunction by blunting oxidative stress, COX-2 levels and increasing adiponectin levels in hypertensive rats on HF diet.

## Background

Obesity and hypertension are two major risk factors that lead to increased incidence of cardiac diseases including coronary artery disease, heart failure and cardiomyopathy [1-3]. Blood pressure, which strongly correlates with body mass index, is one of the most important determinants of cardiovascular function [4]. In addition, obesity also leads to abnormal cardiac function through mechanisms that are independent of hypertension [5,6]. Metabolic syndrome is a clinico-pathological condition which entails superimposition of these abnormalities and is characterized by systemic inflammation and oxidative stress [3,7] A combination of these risk factors leads to disruption of metabolic homeostasis and may further contribute towards progressive cardiovascular dysfunction.

The heme-HO system, comprising of HO-1 (inducible) and HO-2 (constitutive) isoforms, is one of the key defense mechanisms against oxidative stress [8]. This effect of HO system is attributable, in large part, to the antioxidant and anti-apoptotic properties of the heme degradation products, bilirubin/biliverdin and carbon monoxide (CO) [9]. Previous studies have shown that upregulation of HO-1 exerts a cardio protective effect in hypertensive rats [10-14] by reducing myocardial hypertrophy, oxidative stress and inflammation. Over expression of HO-1 is also known to cause adipose tissue remodeling by increasing adiponectin in obese and non-obese diabetic rats and mice [15-18] along with obesity associated suppression of inflammatory cytokines. Adiponectin is an adipose tissue-specific protein that has been shown to have antiatherogenic, antihypertensive and insulin-sensitizing properties [19-21]. An inverse relationship exists between plasma adiponectin levels and systolic blood pressure as well as vascular dysfunction in obese subjects and animals [19,22]. HO-1 functions as a stress response/chaperone protein and increases adiponectin levels which may cause activation of AMPK-AKT signaling [23-25], which contributes to improved NO bioavailability, vascular function, glucose transport and fatty acid oxidation [26,27]. Thus, alterations in the heme-HO system not only influence vascular function but also modulate metabolic and cardiovascular processes which, in turn, are dependent upon activation of adiponectin/AMPK pathways.

The beneficial role of HO enzyme system in animal models of obesity and hypertension are clearly defined but paucity of evidence exists regarding similar effects in co-morbid conditions such as hypertension and obesity. In light of this evidence, the aim of this novel study was to explore the potential effect of HO-1 induction in spontaneously hypertensive rats (SHR) fed a high fat diet, a phenotype designed to mimic metabolic syndrome. We tested our hypothesis by using a well-described high fat regimen [28] that does not cause atherosclerotic lesion formation in mice [29], to address the effects of a known HO-1 inducer, cobalt protoporphyrin (CoPP). To verify that the effects of CoPP were due to an increase in HO-activity, we also treated a group of SHR concurrently with stannous mesoporphyrin (SnMP) to inhibit HO activity. Our results show that obesity exacerbates myocardial and vascular damage in SHRs, and HO-1 induction improves heart function in parallel with increased adiponectin levels and reduced expression of myocardial pro-inflammatory enzymes such as COX-2 and iNOS. Thus, HO-1 appears to play a critical role in the cellular defense against obesity-induced cardiovascular dysfunction in a hypertensive animal model fed a high fat diet. These findings may have important clinical implications in the management of patients with metabolic syndrome.

## Methods

### Animal treatment

All animal studies were approved by the New York Medical College Animal Care and Use Committee in accordance with the National Institutes of Health Guidelines for Care and Use of Laboratory Animals. Fifty-eight seven-week-old male SHRs were purchased from Charles River Laboratories and were divided into four groups: A) SHR control, B) SHR-fat, C) SHR-fat and CoPP treatment, D) SHR-fat and CoPP and SnMP treatment. SHR rats were fed ad libitum either with a normal diet (group A) containing 11% fat, 62% carbohydrate, and 27.0% protein total, 12.6 KJ/g or a high fat diet (groups B, C, D) containing 58% fat from lard, 25.6% carbohydrate, and 16.4% protein yielding 23.4 KJ/g (Bio-SERV, Frenchtown, NJ) for 15 weeks [28,30]. The diet used is distinct from the so-called "Western" or "atherosclerotic" diet which contains, in addition to high fat, cholesterol and bile acids. While the high fat diet used in the present study results in obesity, it does not cause atherosclerotic lesion formation in mice [29]. After 4 weeks of high fat diet, cobalt protoporphyrin (CoPP), an inducer of HO-1, was administered intraperitoneally once a week (3 mg/kg) for 11 weeks to SHR rats maintained on a high fat diet. Some of the SHR treated with CoPP were concurrently treated with tin mesoporphyrin IX dichloride (SnMP), to inhibit HO activity, which was administered intraperitoneally three times a week (20 mg/kg) [11] to ascertain that any effects of CoPP treatment were related to increased HO activity. The untreated SHR rats maintained on the high fat diet were administered the vehicle for CoPP and SnMP once a week and 3 times a week respectively (0.1 mM sodium citrate buffer pH 7.8) for 11 weeks.

Rats were weighed every 7 days and systolic blood pressure was determined weekly by the tail-cuff method.

After a 6-hour fast, rats were anesthetized with sodium pentobarbital (65 mg/kg, i.p.) and blood was obtained from a tail vein for glucose measurement using a glucometer (Lifescan Inc., Miligitas, CA). Blood samples were then collected and stored as previously described [15].

### Isolated Heart Preparation

Three days after the last CoPP (or vehicle) injection, rats were anaesthetized with pentobarbital, i.p., and heparinized via the left femoral vein (250 units/kg). The heart was rapidly excised, placed in cold perfusion medium and weighed. The isolated hearts were attached to the Langerdorff apparatus and retrogradely perfused (at 37°C) using constant perfusion pressure of 80 cm H_2_O, then perfusion pressure was decreased to 20 mmHg for 30 min, and then pressure was increased back to 80 mmHg for the remaining 30 min (reperfusion) [29]. The perfusion medium consisted of oxygenated Krebs-Henseleit buffer [31,32]. For measurement of ventricular systolic and end diastolic pressure (EDP), latex balloons were inserted into the left ventricle of the heart through the mitral valve and connected to a Harvard pressure transducer. In each experiment EDP was set at 10 mmHg and kept stable during the first 10 minutes of perfusion. Coronary perfusion pressure (CPP) was monitored by a second pressure transducer connected to the aortic cannula. Left ventricular developed pressure (LVDevP), EDP, dP/dTmax and dP/dTmin were all derived or calculated from the continuous monitoring of the LV pressure signal. In all experiments, coronary flow was continuously monitored by collecting the cardiac effluent. Coronary resistance (CR) was defined as input pressure divided by coronary flow per gram of myocardial tissue (mmHgxminxg/mL). At the end of each experiment, hearts were collected, half were used for histology examination and half of them were rapidly frozen in liquid nitrogen and stored at -80°C.

### Assessment of Myocyte Cross-Sectional Area, Myocardial Fibrosis and Collagen in Myocardial Tissue

Hearts were fixed in 10% buffered formalin, and embedded in paraffin wax and sectioned to 5 μm. For measurement of the cross-sectional area, 100 cells (per animal) from the left ventricular wall were randomly chosen and analyzed in hematoxylin staining. The myocyte cross-sectional area and myocardial fibrosis were quantitatively analyzed with Image Pro-Plus 4.5.1 software in digitalized microscopic images. Myocardial fibrosis in the tissue sections was quantitatively analyzed by morphometry in 2 ways: (1) on the perivascular fibrosis, and (2) on myocardial tissue (total fibrosis index). The collagen in myocardial tissue was visualized by Sirius Red staining under polarization microscopy and then quantified.

### Assessment of Vascular Reactivity

The aorta was removed, cleaned of fat and loose connective tissue, placed in cold Krebs-bicarbonate solution, and sectioned into 3-mm-long rings. Vasorelaxation responses of phenylephrine-constricted arteries to cumulative increments in acetylcholine (10^-9 ^to 10^-4 ^mol/L) were examined in the presence of indomethacin (10 μmol/L) as described [33].

### Western Blot Analysis of Cardiac Tissue for protein expression

At the time of sacrifice, hearts were harvested, and stored at -140°C. Frozen hearts were pulverized under liquid nitrogen and placed in a homogenization buffer prior to immunoblotting with antibodies against HO-1, and HO-2 (Stressgen Biotechnologies Corp., Victoria, BC), COX-2, TX synthase, NOX-2, AKT, AMPK, pAMPK(Thr172), pAKT and adiponectin (Cell Signaling Technology, Inc., Beverly, MA) and eNOS, peNOS(serine 1177), and iNOS (Santa Cruz Biotechnology, Santa Cruz, CA). Immunoblotting was performed in cardiac tissue as previously described [15,33].

### Measurement of HO activity

HO activity in heart tissue was assayed as described previously [15] using a technique in which bilirubin, the end product of heme degradation, was extracted with chloroform, and its concentration was determined spectrophotometrically (dual UV-visible beam spectrophotometer Lambda 25; PerkinElmer Life and Analytical Sciences, Waltham, MA) using the difference in absorbance at a wavelength from 460 to 530 nm, with an extinction coefficient of 40 mM^-1 ^cm^-1^.

### Measurements of O_2_^- ^production and total cholesterol levels

Total cholesterol was measured in serum using a cholesterol Quantification Kit (Biovision, Mountainview, CA) according to the manufacturer's instructions. For the detection of O_2_^-^, homogenized hearts were placed in plastic scintillation vials containing 5 μmol/l lucigenin in a final volume of 1 ml of air-equilibrated Krebs solution as described previously [15].

### Plasma Adiponectin and inflammatory cytokines Measurements

The high molecular weight (HMW) HMW form of adiponectin, IL-6, TNF-α and TXB2 levels were determined using an ELISA assay (Pierce Biotechnology, Inc., Woburn, MA) as described previously [15].

### Statistical Analysis

The data are presented as mean ± standard error (SEM) where n = 6/group for the results. For comparison between treatment groups, the Null hypothesis was tested by a single factor analysis of variance (ANOVA) for multiple groups or unpaired *t-*test for two groups. Statistical significance (p < 0.05) between the experimental groups was determined by the Fisher method of analysis for multiple comparisons.

## Results

### Effect of a high-fat diet on body weight and metabolic response

**Figure 1:**
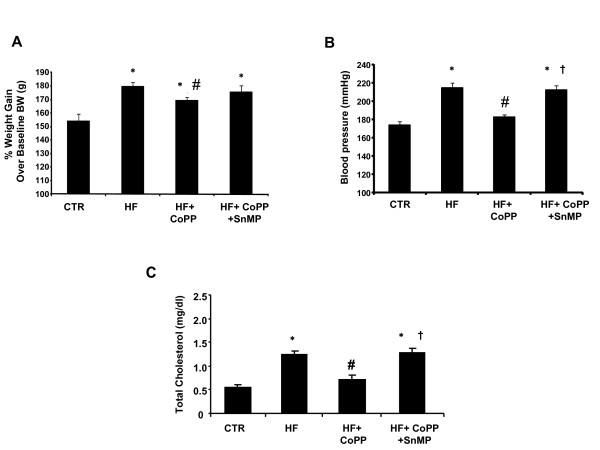
**Effect of a high fat diet and treatment with CoPP and CoPP + SnMP in SHR (n = 14 per group) on **A) **% of body weight gain**. *p < 0.01 vs. control, p < 0.05 vs. high fat **B) **Blood pressure, which was measured by tail cuff method. *p < 0.01 vs. control, #p < 0.01 vs. HF, †p < 0.01 vs. HF+ CoPP. **C) **plasma total cholesterol levels. *p < 0.05 vs. control, #p < 0.05 vs HF, †p < 0.05 vs HF+CoPP.

Figure 1A shows the percent change in body weight over its baseline values in the 4 groups. In untreated SHR rats body weight increased 54% ± 5.5 on a normal diet over a period of 15 weeks, whereas in rats fed a high fat diet body weight increased 79% ± 3.7 (p < 0.05). The total body weight observed after 15 weeks of study was 367 ± 10.7 gms in SHR controls and 419 ± 6.3 gms in SHR rats fed a high fat diet (data not shown). We also examined the effect of long-term CoPP treatment on body weight gain in response to a high fat diet. Weekly treatment with CoPP was started 4 weeks after the initiation of the high fat diet and was well tolerated by the SHR (n = 14/group); activity and grooming were maintained during CoPP treatment. Rats fed a high fat diet and concurrently exposed to CoPP, showed reduction in body weight as compared to SHR rats on high fat diet, 68% ± 2.4 (p < 0.05). A significant increase in body weight was seen when animals fed a high fat diet were exposed to CoPP + SnMP. The weight gain was 75% ± 4.9 and was not significantly different from animals fed a high fat diet. The total body weight observed after 15 weeks of study in rats fed a high fat diet and concurrently exposed to CoPP was 386 ± 9.7 gms and was increased to 416 ± 8.1 gms in SHR rats fed a high fat diet and treated with CoPP and SnMP (data not shown).

Systolic blood pressure was increased over the 15-week period in SHR rats (Figure 1B; n = 6/group). The systolic blood pressure was 175 ± 11 mmHg in the SHR control and was significantly increased in the rats fed a high fat diet, 211 ± 9 mmHg (p < 0.05). The elevation in systolic pressure was attenuated by CoPP treatment in SHR fed a high fat diet whereas SnMP treatment nullified the antihypertensive effect of CoPP in SHR fed a high-fat diet (Figure 1B). The mean blood glucose level in the SHR rats maintained on a normal diet was 128 ± 4 mg/dl, and was increased to 173 ± 14 mg/dl by a high fat diet (*p *< 0.05; n = 6/group) (data not shown). This increase in blood glucose levels was significantly attenuated by CoPP treatment in SHR rats fed a high fat diet (137 ± 4.5 mg/dl) and this effect was reversed by treatment with SnMP (180 ± 7.8 mg/dl) (data not shown).

Plasma cholesterol levels remained elevated in SHRs fed a high-fat diet as compared to their controls. Plasma cholesterol levels were 0.55 ± 0.11 in SHRs fed a normal diet for 15 weeks, and levels were increased to 1.25 ± 0.15 mg/dL by 15 weeks on the high-fat diet (*P *< 0.05) (Figure 1C). CoPP treatment prevented the increase in cholesterol levels in SHR while concomitant treatment with SnMP blocked the effect of CoPP.

### Effect of high fat diet on cardiac parameters

**Figure 2:**
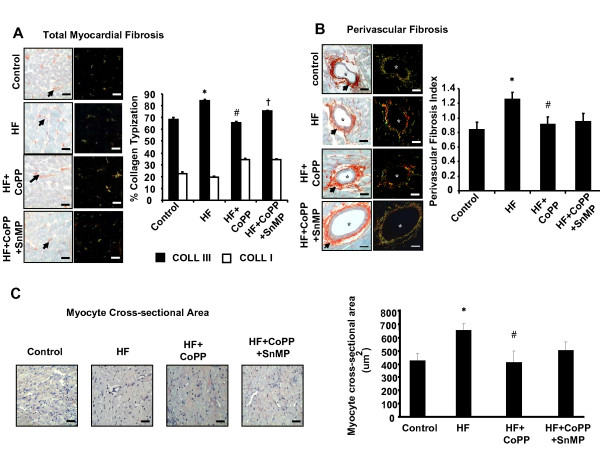
**Histology change of the heart**. **A) **Total myocardial fibrosis. Transverse section of left ventricle stained with Sirius Red and observed under light and polarized microscope. Whole collagen is red stained and indicated by arrows. Type I collagen is green-yellow, while Type III collagen is orange-red. Bar. 50 um. *p < 0.01 vs. control, p < 0.05 vs. HF, † p < 0.05 vs HF+ CoPP **B) **Perivascular fibrosis. Transverse sections of intramuscular arteries with perivascular fibrosis stained with Sirius Red and observed under light and polarized microscope. Bar. 100 μm. *p < 0.01 vs. control, #p < 0.05 vs.HF. **C) **Myocyte cross-sectional area. Left ventricular myocyte cross-sectional areas stained with hematoxylin. The myocyte cross-sectional area and myocardial fibrosis were quantitatively analyzed with Image Pro-Plus 4.5.1 software in digitalized microscopic images. For measurement of the cross-sectional area, 100 cells (per animal) from the left ventricular wall were randomly chosen and analyzed in hematoxylin staining. Bar. 50 μm. *p < 0.01 vs. control, #p < 0.05 vs. HF.

The collagen III was higher in hearts of SHRs fed a high fat diet (*P *< 0.05) when compared to untreated animals (Figure 2A). The perivascular fibrosis index was higher in SHRs fed a high fat diet than those animals fed a normal diet (*P *< 0.05) (Figure 2B). CoPP administration prevented the occurrence of these increases in animals fed a high fat diet on perivascular fibrosis while concurrent administration of SnMP did not significantly reversed the effect of CoPP(Figure 2B). The myocyte cross-sectional area was increased by a high fat diet in SHRs. CoPP treatment prevented the increase in myocyte cross-sectional area while concurrent administration of SnMP did not significantly reversed the effect of CoPP (Figure 2C).

### Effect of high fat diet on CR and cardiac function during ischemia/reperfusion

**Figure 3:**
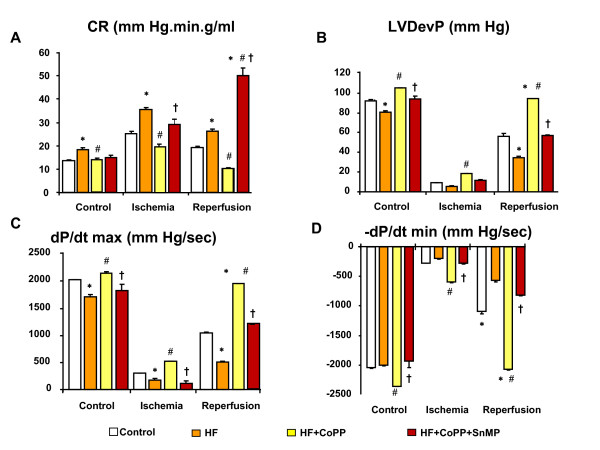
**Effect of a high fat diet, CoPP and SnMP on coronary resistance and LV function**. Isolated heart from control SHR rats after HF diets, HF rats, HF+CoPP and HF+CoPP+SnMP were studied in Langerdorff configuration with a protocol of ischemia/reperfusion. Coronary resistance (CR) (Figure 3A), left ventricular developed pressure (LVDevP) (Figure 3B), dP/dtmax(Figure 3C) and dP/dtmin (Figure 3D) in each stage of ischemia/reperfusion, i.e. baseline (bas), low pressure perfusion (Low P i.e. ischemia) and high pressure i.e.reperfusion (Rep) in SHRs, The results are means ± SE, *p < 0.05 vs. control, # p < 0.05 vs. HF, †p < 0.05 vs. HF+ CoPP.

Our results show that during low perfusion pressure (i.e. ischemia), CR increased over baseline values in all groups, but CR in SHR mice was significantly higher than in controls (p < 0.05)(Figure 3A). This phenomenon, defined as 'paradoxical vasoconstriction', has been described previously by our group in both control and diabetic animals [34]. CoPP modulated coronary tone during the ischemic period significantly reducing vasoconstriction. After 30 min of reperfusion, CR was still significantly increased over baseline values in high fat hearts (p < 0.05), while CR in High fat CoPP group returned to baseline values (Figure 3A). The CoPP-"normalization" of coronary tone at reperfusion in HF hearts was mirrored by better overall cardiac function during both low pressure ischemia and reperfusion times. Indeed, LVDevP (Figure 3B), dP/dtmax (Figure 3C) and dP/dtmin (Figure 3D) were all significantly improved compared to the untreated group (p < 0.05).

### Effect of high fat diet on Vascular Reactivity and superoxide levels

**Figure 4:**
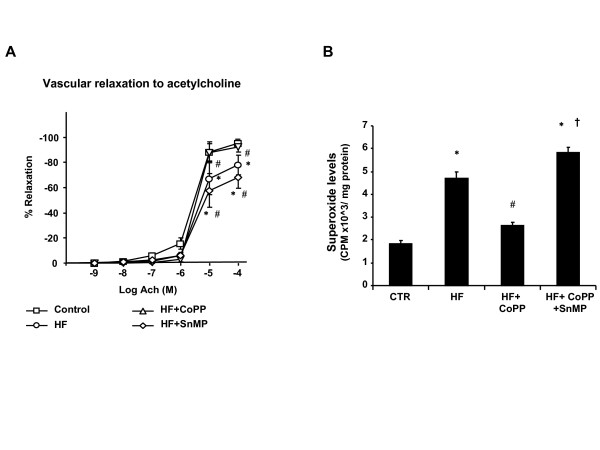
**Effect of a high fat diet, CoPP and SnMP on **A) **vascular reactivity in SHR rats**. Aorta was precontracted with phenylephrine (PE) and then exposed to acetylcholine (Ach) in a dose dependent manner (10^-9 ^-10^-4 ^M). *p < 0.05 vs. control, #p < 0.05 vs. HF+ CoPP. **B) **Superoxide production. * p < 0.01 vs. control, # p < 0.01 vs. HF, † p < 0.01 vs. HF+ CoPP.

Aortic endothelial dilatory responses to acetylcholine (at concentration of 10^-5 ^and 10^-4 ^mmol/L respectively) were significantly impaired in SHRs after 15 weeks of a high-fat diet compared with those fed a normal diet (*P *< 0.05) (Figure 4A). Endothelial function was improved in SHRs as a result of the CoPP treatment (*P *< 0.05), but exacerbated by SnMP (Figure 4A) indicating that it is specifically the endothelial dilatory response that is impaired by a high fat diet in this animal model. Cardiac oxidative stress was increased as cortical superoxide generation was greater in SHR fed a high fat diet compared with rats fed a normal diet (Figure 4B where n = 6/group), (p < 0.05). CoPP treatment prevented the increase in cardiac O_2_^- ^generation in SHR maintained on a high fat diet (p < 0.01), an effect abolished by concurrent administration of SnMP.

### Effect of high fat diet on plasma adiponectin, inflammatory cytokines and TxB2 Levels

**Figure 5:**
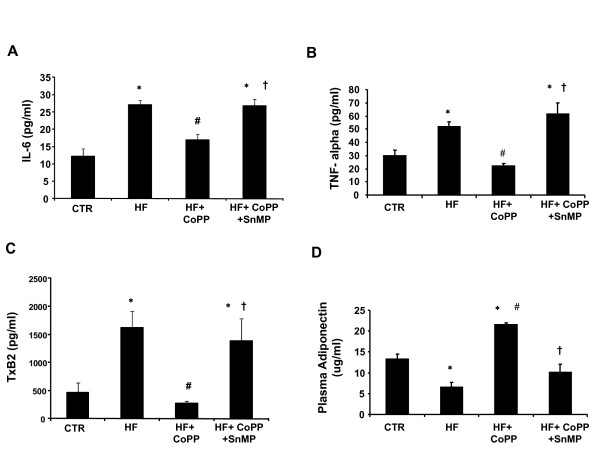
**Effect of a high fat diet, CoPP and SnMP on plasma cytokines, TXB2 and adiponectin levels in SHR animals**. n = 6 for each group. **A) **Plasma IL-6 levels. * p < 0.01 vs. control, # p < 0.01 vs. HF, † p < 0.01 vs. HF+ CoPP. **B) **TNF-α levels. *p < 0.05 vs. control, #p < 0.05 vs. HF, †p < 0.05 vs. HF+ CoPP. **C) **TxB2 levels. *p < 0.05 vs. control, #p < 0.05 vs. HF, †p < 0.05 vs. HF+ CoPP. **D) **adiponectin levels. *p < 0.05 vs. control, #p < 0.05 vs. HF, †p < 0.05 vs. HF+ CoPP.

Plasma IL-6 and TNF-α (Figure 5A and 5B) levels were greater in SHR fed a high fat diet compared to rats fed a normal diet (n = 6/group),(p < 0.05). Increasing HO-1 by CoPP administration significantly decreased plasma cytokines and this effect was prevented by concurrent SnMP treatment (p < 0.01, Figure 5A and 5B). Similar pattern was observed in plasma TxB2 levels as shown in Figure 5C (n = 6/group), (p < 0.05). Plasma adiponectin levels were lower in rats fed a high fat diet when compared to control animals fed a normal diet (p < 0.05; n = 6/group) (Figure 5D). This effect was reversed when rats were treated with CoPP (p < 0.05). Indeed, in SHR rats maintained on a high-fat diet and treated with CoPP, plasma adiponectin levels were higher than those in the respective control groups (p < 0.05). Concurrent administration of SnMP with CoPP in the SHR fed a high fat diet prevented the increase in adiponectin, so that the levels of this protein were not different from those in the untreated SHR.

### Effect of high fat diet on Cardiac COX-2, TxA2 and NOX-2 Levels

**Figure 6:**
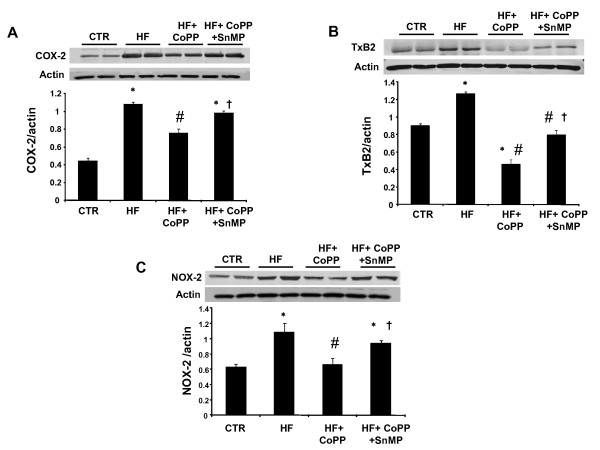
**Western blot and densitometry analysis of **A) **COX-2; **B) **TXB2; **C) **NOX-2 in hearts obtained from SHR**. Rats were fed a high fat diet and treated with CoPP and CoPP + SnMP. Data are shown as the COX-2/actin ratio, TXB2/actin ratio and NOX-2/actin ratio, respectively. n = 6, *p < 0.05 vs. control, #p < 0.05 vs. HF, †p < 0.05 vs. HF+ CoPP.

Hearts isolated from SHRs fed a high fat diet showed a significant increase in markers of oxidative stress compared to animals fed a normal diet (p < 0.05, respectively) (Figures 6A, B and 6C). Treatment with CoPP resulted in a decrease in COX-2, TxA2 and NOX-2 expression in SHRs fed a high fat diet (p < 0.01 respectively), an effect abolished by concurrent administration of SnMP.

### Effect of high fat diet on cardiac HO-1

**Figure 7:**
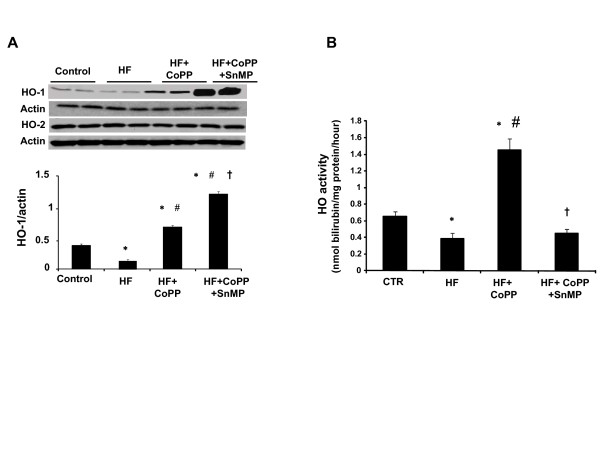
**A) Western blot and densitometry analysis of HO-1 and HO-2 proteins in hearts from SHR**. Rats were fed a high fat diet and treated with CoPP or CoPP + SnMP. Immunoblots were performed with antibodies against rat HO-1 and HO-2. Data are shown as mean band density normalized relative to β-actin. n = 6, *p < 0.01 vs. control, #p < 0.01 vs. high fat, †p < 0.01 vs. high fat CoPP. **B) **Cardiac HO activity of SHR and those fed a high fat diet and treated with CoPP or CoPP + SnMP. n = 6 for each group. *p < 0.01 vs. control, #p < 0.01 vs. high fat, †p < 0.01 vs. high fat CoPP.

First, we confirmed that CoPP treatment for 11 weeks resulted in up-regulation of HO-1. HO-1 protein in the hearts of SHR fed a high fat diet was significantly less than that of the respective control group (Figure 7A where n = 6/group) when the latter was fed a normal diet (p < 0.05). Treatment with CoPP resulted in a significant increase in HO-1 levels in SHR fed a high-fat diet. Although SnMP treatment showed a significant increase in HO-1 expression (Figure 7A), it is a potent inhibitor of HO activity as shown previously [11,35] and thus prevents heme degradation and inhibits formation of CO and biliverdin. HO-2 levels were unaffected either by high fat diet or by CoPP treatment (Figure 7A). Consistent with protein expression, HO activity was significantly decreased in obese SHR hearts compared to the control group (Figure 7B). CoPP treatment significantly increased HO activity in SHR fed a high fat diet, 1.45 ± 0.20 nmol bilirubin/mg/hr compared to 0.39+0.09 nmol bilirubin/mg/hr in untreated SHR fed a high fat diet (p < 0.001). The concurrent administration of SnMP resulted in significant decrease of HO activity as shown in Figure 7B.

### Effect of high fat diet on Cardiac adiponectin, pAMPK and pAKT Expression

**Figure 8:**
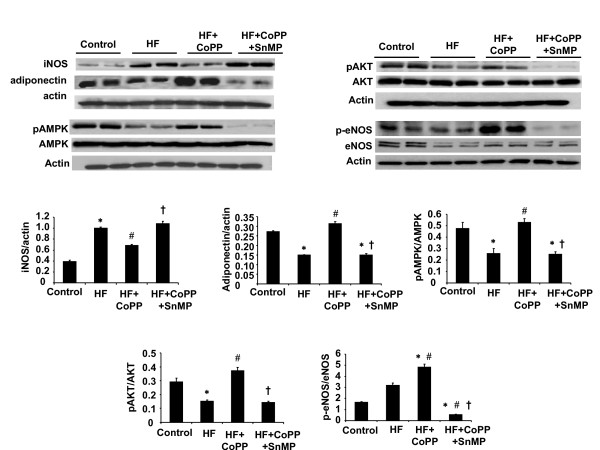
**Western blot and densitometry analysis of adiponectin, AMPK, pAMPK, AKT, pAKT, eNOS, p-eNOS, iNOS proteins in heart from SHRs**. Data are shown as mean band density normalized relative to β-actin or pAMPK/AMPK ratio or pAKT/AKT or p-eNOS/eNOS ratio. n = 6, * p < 0.05 vs. control, #p < 0.05 vs. HF, †p < 0.05 vs. HF+ CoPP.

Cardiac adiponectin levels, normalized against β-actin, exhibited a similar pattern to plasma adiponectin levels. Thus, feeding SHR a high fat diet for 15 weeks resulted in a decrease in adiponectin compared to untreated SHR (Figure 8; n = 6/group). Induction of HO-1 with CoPP increased cardiac adiponectin levels in hypertensive rats (p < 0.01) and the increase in SHR was prevented and reversed to a decrease when the rats were, also, treated with SnMP to inhibit HO activity (Figure 8). A high fat diet resulted in significant decreases in pAMPK and pAKT expression in hearts from SHR (p < 0.05; n = 6/group) (Figure 8). CoPP administration caused a significant increase in the expression of pAKT and pAMPK in the rats fed a high fat diet (p < 0.05) compared to untreated rats fed a high fat diet. The changes in expression of pAMPK and pAKT paralleled those seen with HO-1 protein expression. In SHR maintained on a high fat diet and treated with CoPP, the concurrent administration of SnMP prevented the increase in pAKT and pAMPK; indeed, the expression of both pAKT and pAMPK was reduced to levels lower than those seen in SHR on the high fat diet alone (p < 0.01).

### Effect of high fat diet on Cardiac eNOS, peNOS and iNOS Levels

Compared to animals fed a normal diet, SHR animals fed a high fat diet exhibited lower levels of eNOS and peNOS protein (p < 0.05) (Figure 8) CoPP administration produced an enhanced expression of eNOS and peNOS protein (p < 0.05 compared to untreated animals) in SHRs fed a high fat diet (Figure 8). In contrast, SnMP administration resulted in eNOS and peNOS protein in SHRs fed a high fat diet (Figure 8). Hearts isolated from SHRs fed a high fat diet showed a significant increase in iNOS expression compared to animals fed a normal diet (p < 0.05, respectively) (Figures 8). Treatment with CoPP resulted in a decrease in iNOS in SHRs fed a high fat diet (p < 0.0, Figure 8). In contrast, SnMP did not prevent the increase of iNOS expression in SHRs fed a high fat diet (Figures 8).

## Discussion

The results of the present study demonstrate that SHR fed a high fat diet develop patho-physiological abnormalities similar to that observed in metabolic syndrome. This phenotype is characterized by increased levels of body weight, blood cholesterol and blood pressure along with an accelerated decline in cardiac function when compared to SHR maintained on a normal diet. We, also, demonstrated that cardiac HO-1 induction, accompanied by increased plasma and tissue adiponectin levels, resulted in the improvement of cardiovascular function as manifested by a decrease in blood pressure, coronary resistance (CR), myocardial fibrosis; and increase in left ventricular function and vascular relaxation, as compared to control. The upregulation of HO-1 was associated with a concomitant decrease in the levels of O_2_^-^, COX-2 and iNOS, markers of oxidative stress. Furthermore, there was a decrease in cardiac remodeling, and an increase in the expression of cardiac pAKT, pAMPK and peNOS via induction of HO-1-adiponectin axis. To the best of our knowledge, this is the first report showing a protective effect of HO-adiponectin axis in a co-morbid condition where a pre-existing cardio-vascular pathology is further aggravated by addition of a HF diet.

High fat intake increased body weight, serum cholesterol and blood pressure in SHR and these changes in metabolic indices were associated with cardiovascular dysfunction in these animals. Previous studies have shown that HO-1 induction decreases obesity, reduces levels of visceral and subcutaneous fat and normalizes the metabolic profile in obese rats and mice [15,17,36,37]. Also HO-1 overexpression is known to improve cardiovascular dysfunction in hypertensive rats [7,11]. In contrast, in the current study we induced a metabolic syndrome-like phenotype in hypertensive animals. SHR demonstrate chronic hypertension, oxidative stress and cardiac damage [38]. All of these parameters were worsened by the addition of high fat diet, strengthening our hypothesis that obesity and the associated metabolic abnormalities accelerate pathological pre-existing cardiovascular changes. Reversal of these patho-physiological abnormalities by HO-1-adiponectin induction corroborates the protective effects of the heme-oxygenase system in such a setting.

Metabolic syndrome-mediated increases in oxidative stress contribute to cardiovascular dysfunction via endothelial cell sloughing and beta cell apoptosis [39]. Sustained increases in O_2_^- ^levels and cytokines, including TNF-α and its receptor, lead to monocyte phenotype transition, myocytic apoptosis, and activation of matrix metalloproteinase. This, in turn, modifies the interstitial matrix, augmenting further ventricular remodeling [40,41]. COX-2 is considered a pro-inflammatory enzyme as free radicals and prostaglandins (PGs) are produced during its catalytic cycle [8]. It has been shown in our previous reports that upregulation of HO-1 decreases vasoconstrictors, such as cyclooxygenase (COX-2), PGs and thromboxane syntheses (TxA2) levels [8,42] by regulating the cellular heme levels and ROS. The heme-HO system is a stress response system (reviewed in [8] that undergoes activation under conditions of increased oxidative stress such as those presented here. Induction of HO-1 resulted in decreased cardiac levels of superoxide and NOX-2 expression which may be due to a decrease in the levels of NADPH oxidase [43], a heme-dependent protein, and/or an increase in the levels of superoxide dismutase EC-SOD [44]. Also in the present study, increased cardiac iNOS expression and impaired vascular relaxation in rats fed a high-fat diet was reversed by HO-1 induction which may involve the interplay of one of the various mechanisms including, CO generation, HO-1-induced increase in eNOS expression and increased NO bioavailability due to an increase in cellular antioxidants [37,45-47].

In the present study, a decrease in coronary vascular reactivity manifested by coronary resistance, myocardial fibrosis and cardiac function was found in SHRs fed a high fat diet. The increase in expression of HO-1/adiponectin reverses these deleterious effects with a resultant improvement in energy metabolism and an amelioration of the damaged endothelial and cardiac function seen in SHRs fed a high fat diet. We studied coronary microvascular reactivity and hemodynamics in the isolated, empty, beating heart of SHRs fed a high fat diet. This was prevented in CoPP-treated animals by SnMP suggesting the seminal role of increased HO activity in instigating the changes attributable to increased HO-I expression. This finding highlights the role of the HO system in the preservation of microvascular and cardiac function.

Apart from effects on heme degradation products, HO1 up-regulation was associated with increased cardiac and plasma levels of adiponectin. This causality between HO activity and adiponectin release was strengthened by the inhibitory effects of SnMP on both HO activity and adiponectin levels. It has been recently shown that the beneficial effects of heme- HO system in established cardiovascular-metabolic disorders is mediated, at least in part, via its effect on adiponectin-dependent pathways [15,48,49]. Results presented in the current study support and advance our hypothesis that, in addition to its antioxidant properties, the heme-oxygenase system enhances the adiponectin axis which, in turn, modulates multiple physiological processes and may contribute towards HO-mediated attenuation of cardiac dysfunction [17,18,50].

The HO-1-mediated increase in adiponectin was associated with an increase in cardiac pAMPK-pAKT signaling and cross-talk between AMPK and AKT levels appear to correlate with HO-1 and adiponectin levels [16,18,25,51]. This is of particular importance in the setting of myocardial ischemia of SHR rats fed a high fat diet due to the very-high-energy demands and low-energy reserves of the heart. Amplifying signaling through AMPK by HO-1 induction during early reperfusion is beneficial to the injured myocardium due to the ability of AMPK to promote ATP generation [52,53] and to attenuate cardiomyocyte apoptosis [54]. An increase in AMPK-AKT signaling is considered an important metabolic response that is necessary for the attenuation of ROS-mediated cardiac and endothelial dysfunction [55] and both pAMPK and pAKT use eNOS as a substrate and enhance the levels of peNOS [8,56,57]. The results of this study support this link as induction of HO-1-adiponectin axis, also, increased peNOS expression in the heart of SHR. The seminal role of increased HO-1 expression and HO activity in cardiac protection is further strengthened by the results obtained when SnMP was concurrently administered with CoPP; the inhibition of HO activity prevented the beneficial effects of HO-1 induction in obese SHR with regard to blood pressure, adiponectin, pAKT and pAMPK. In summary, these observations support the beneficially role of pharmaco-genetic interventions targeted towards HO-1-adiponectin axis in patients with metabolic syndrome. Such patients often exhibit chronic energy imbalance along with a wide array of cardiovascular abnormalities amenable to aggravation by confounding factors such as diet induced obesity. Restoration of metabolic homeostasis by activation oh HO-1-adiponectin axis could not only improve the energy profile but also attenuate associated cardiovascular patho-physiological alterations observed in the patients with metabolic syndrome.

## Conclusion

In conclusion, the results of the present study demonstrate that upregulation of HO-1 in association with increased levels of adiponectin prevents vascular and cardiac dysfunction in SHRs fed a high fat diet, a phenotype designed to mimic metabolic syndrome. The pharmacological enhancement of HO-1 expression, resulting in a phenotype resistant to injurious stimuli, permits the heart to initiate a crucial and immediate defense against the events associated with the metabolic syndrome, thereby preventing the continued deterioration in cardiac function associated with this disease.

## Competing interests

The authors declare that they have no competing interests.

## Authors' contributions

*JC and KS contributed equally to this work

JC drafted the manuscript. KS performed all the experiments except vascular activity. SRM did the vascular activity. RR carried out the morphological studies in heart. NGA conceived the study, and participated in its design and coordination.

All authors read and approved the final manuscript.

## References

[B1] Zalesin KC Franklin BA Miller WM Peterson ED McCullough PA Impact of obesity on cardiovascular disease Med Clin North Am 2011 95 919 937 10.1016/j.mcna.2011.06.005 21855700

[B2] Hall JE The kidney, hypertension, and obesity Hypertension 2003 41 625 633 10.1161/01.HYP.0000052314.95497.78 12623970

[B3] Knight SF Quigley JE Yuan J Roy SS Elmarakby A Imig JD Endothelial dysfunction and the development of renal injury in spontaneously hypertensive rats fed a high-fat diet Hypertension 2008 51 352 359 10.1161/HYPERTENSIONAHA.107.099499 18158349 PMC2491336

[B4] Garrison RJ Kannel WB Stokes J Castelli WP Incidence and precursors of hypertension in young adults: the Framingham Offspring Study Prev Med 1987 16 235 251 10.1016/0091-7435(87)90087-9 3588564

[B5] Kenchaiah S Evans JC Levy D Wilson PW Benjamin EJ Larson MG Kannel WB Vasan RS Obesity and the risk of heart failure N Engl J Med 2002 347 305 313 10.1056/NEJMoa020245 12151467

[B6] Mottillo S Filion KB Genest J Joseph L Pilote L Poirier P Rinfret S Schiffrin EL Eisenberg MJ The metabolic syndrome and cardiovascular risk a systematic review and meta-analysis J Am Coll Cardiol 2010 56 1113 1132 10.1016/j.jacc.2010.05.034 20863953

[B7] Berg AH Scherer PE Adipose tissue, inflammation, and cardiovascular disease Circ Res 2005 96 939 949 10.1161/01.RES.0000163635.62927.34 15890981

[B8] Abraham NG Kappas A Pharmacological and clinical aspects of heme oxygenase Pharmacol Rev 2008 60 79 127 10.1124/pr.107.07104 18323402

[B9] Wu L Wang R Carbon monoxide: endogenous production, physiological functions, and pharmacological applications Pharmacol Rev 2005 57 585 630 10.1124/pr.57.4.3 16382109

[B10] Sacerdoti D Escalante B Abraham NG McGiff JC Levere RD Schwartzman ML Treatment with tin prevents the development of hypertension in spontaneously hypertensive rats Science 1989 243 388 390 10.1126/science.2492116 2492116

[B11] Botros FT Schwartzman ML Stier CT Jr Goodman AI Abraham NG Increase in heme oxygenase-1 levels ameliorates renovascular hypertension Kidney Int 2005 68 2745 2755 10.1111/j.1523-1755.2005.00745.x 16316349

[B12] Sabaawy HE Zhang F Nguyen X Elhosseiny A Nasjletti A Schwartzman M Dennery P Kappas A Abraham NG Human heme oxygenase-1 gene transfer lowers blood pressure and promotes growth in spontaneously hypertensive rats Hypertension 2001 38 210 215 11509478 10.1161/01.hyp.38.2.210

[B13] Buja LM Myocardial ischemia and reperfusion injury Cardiovasc Pathol 2005 14 170 175 10.1016/j.carpath.2005.03.006 16009313

[B14] Cao J Inoue K Li X Drummond G Abraham NG Physiological significance of heme oxygenase in hypertension Int J Biochem Cell Biol 2009 41 1025 1033 10.1016/j.biocel.2008.10.025 19027871 PMC2745554

[B15] Li M Kim DH Tsenovoy PL Peterson SJ Rezzani R Rodella LF Aronow WS Ikehara S Abraham NG Treatment of obese diabetic mice with a heme oxygenase inducer reduces visceral and subcutaneous adiposity, increases adiponectin levels, and improves insulin sensitivity and glucose tolerance Diabetes 2008 57 1526 1535 10.2337/db07-1764 18375438

[B16] Li M Peterson S Husney D Inaba M Guo K Terada E Morita T Patil K Kappas A Ikehara S Abraham NG Interdiction of the diabetic state in NOD mice by sustained induction of heme oxygenase: possible role of carbon monoxide and bilirubin Antioxid Redox Signal 2007 9 855 863 10.1089/ars.2007.1568 17508911

[B17] Kim DH Burgess AP Li M Tsenovoy PL Addabbo F McClung JA Puri N Abraham NG Heme oxygenase-mediated increases in adiponectin decrease fat content and inflammatory cytokines, tumor necrosis factor-alpha and interleukin-6 in Zucker rats and reduce adipogenesis in human mesenchymal stem cells J Pharmacol Exp Ther 2008 325 833 840 10.1124/jpet.107.135285 18334666

[B18] Nicolai A Li M Kim DH Peterson SJ Vanella L Positano V Gastaldelli A Rezzani R Rodella LF Drummond G Kusmic C L'Abbate A Kappas A Abraham NG Heme Oxygenase-1 Induction Remodels Adipose Tissue and Improves Insulin Sensitivity in Obesity-Induced Diabetic Rats Hypertension 2009 53 508 515 10.1161/HYPERTENSIONAHA.108.124701 19171794 PMC2745551

[B19] Iwasa Y Otsubo S Ishizuka T Uchida K Nitta K Influence of serum high-molecular-weight and total adiponectin on arteriosclerosis in IgA nephropathy patients Nephron Clin Pract 2008 108 c226 c232 10.1159/000119717 18332637

[B20] Huang KC Chen CL Chuang LM Ho SR Tai TY Yang WS Plasma adiponectin levels and blood pressures in nondiabetic adolescent females J Clin Endocrinol Metab 2003 88 4130 4134 10.1210/jc.2003-030158 12970275

[B21] Iwashima Y Katsuya T Ishikawa K Ouchi N Ohishi M Sugimoto K Fu Y Motone M Yamamoto K Matsuo A Ohashi K Kihara S Funahashi T Rakugi H Matsuzawa Y Ogihara T Hypoadiponectinemia is an independent risk factor for hypertension Hypertension 2004 43 1318 1323 10.1161/01.HYP.0000129281.03801.4b 15123570

[B22] Abraham NG Kruger A Peterson S High serum levels of adiponectin in HO-1preconditioning in mice and rats with Type 2 diabetes improve vascular function American Heart Association 2007 in press

[B23] Cao J Drummond G Inoue K Sodhi K Li XY Omura S Upregulation of Heme Oxygenase-1 Combined with Increased Adiponectin Lowers Blood Pressure in Diabetic Spontaneously Hypertensive Rats through a Reduction in Endothelial Cell Dysfunction, Apoptosis and Oxidative Stress Int J Mol Sci 2008 9 2388 2406 10.3390/ijms9122388 19330083 PMC2635644

[B24] Peterson SJ Drummond G Kim DH Li M Kruger AL Ikehara S Abraham NG L-4F treatment reduces adiposity, increases adiponectin levels and improves insulin sensitivity in obese mice J Lipid Res 2008 49 1658 1669 10.1194/jlr.M800046-JLR200 18426778 PMC2443999

[B25] Peterson SJ Kim DH Li M Positano V Vanella L Rodella LF Piccolomini F Puri N Gastaldelli A Kusmic C L'Abbate A Abraham NG The L-4F mimetic peptide prevents insulin resistance through increased levels of HO-1, pAMPK, and pAKT in obese mice J Lipid Res 2009 50 1293 1304 10.1194/jlr.M800610-JLR200 19224872 PMC2694329

[B26] Hardie DG Minireview: the AMP-activated protein kinase cascade: the key sensor of cellular energy status Endocrinology 2003 144 5179 5183 10.1210/en.2003-0982 12960015

[B27] Hopkins TA Ouchi N Shibata R Walsh K Adiponectin actions in the cardiovascular system Cardiovasc Res 2007 74 11 18 10.1016/j.cardiores.2006.10.009 17140553 PMC1858678

[B28] Schreyer SA Wilson DL LeBoeuf RC C57BL/6 mice fed high fat diets as models for diabetes-accelerated atherosclerosis Atherosclerosis 1998 136 17 24 10.1016/S0021-9150(97)00165-2 9544727

[B29] Molnar J Yu S Mzhavia N Pau C Chereshnev I Dansky HM Diabetes induces endothelial dysfunction but does not increase neointimal formation in high-fat diet fed C57BL/6J mice Circ Res 2005 96 1178 1184 10.1161/01.RES.0000168634.74330.ed 15879311

[B30] Surwit RS Kuhn CM Cochrane C McCubbin JA Feinglos MN Diet-induced type II diabetes in C57BL/6J mice Diabetes 1988 37 1163 1167 10.2337/diabetes.37.9.1163 3044882

[B31] L'Abbate A Neglia D Vecoli C Novelli M Ottaviano V Baldi S Barsacchi R Paolicchi A Masiello P Drummond GS McClung JA Abraham NG Beneficial effect of heme oxygenase-1 expression on myocardial ischemia-reperfusion involves an increase in adiponectin in mildly diabetic rats Am J Physiol Heart Circ Physiol 2007 293 H3532 H3541 10.1152/ajpheart.00826.2007 17906103

[B32] Paolocci N Biondi R Bettini M Lee CI Berlowitz CO Rossi R Xia Y Ambrosio G L'Abbate A Kass DA Zweier JL Oxygen radical-mediated reduction in basal and agonist-evoked NO release in isolated rat heart J Mol Cell Cardiol 2001 33 671 679 10.1006/jmcc.2000.1334 11341236

[B33] Sodhi K Inoue K Gotlinger K Canestraro M Vanella L Kim DH Manthati VL Koduru SR Falck JR Schwartzman ML Abraham NG Epoxyeicosatrienoic acid agonist rescues the metabolic syndrome phenotype of HO-2-null mice J Pharmacol Exp Ther 2009 331 906 916 10.1124/jpet.109.157545 19717790 PMC2784709

[B34] Lara-Castro C Luo N Wallace P Klein RL Garvey WT Adiponectin multimeric complexes and the metabolic syndrome trait cluster Diabetes 2006 55 249 259 10.2337/diabetes.55.01.06.db05-1105 16380500

[B35] Sardana MK Kappas A Dual control mechanism for heme oxygenase: tin(IV)-protoporphyrin potently inhibits enzyme activity while markedly increasing content of enzyme protein in liver Proc Natl Acad Sci USA 1987 84 2464 2468 10.1073/pnas.84.8.2464 3470805 PMC304672

[B36] Dobrian AD Davies MJ Prewitt RL Lauterio TJ Development of hypertension in a rat model of diet-induced obesity Hypertension 2000 35 1009 1015 10775577 10.1161/01.hyp.35.4.1009

[B37] Dobrian AD Davies MJ Schriver SD Lauterio TJ Prewitt RL Oxidative stress in a rat model of obesity-induced hypertension Hypertension 2001 37 554 560 11230334 10.1161/01.hyp.37.2.554

[B38] Bing OH Brooks WW Robinson KG Slawsky MT Hayes JA Litwin SE Sen S Conrad CH The spontaneously hypertensive rat as a model of the transition from compensated left ventricular hypertrophy to failure J Mol Cell Cardiol 1995 27 383 396 10.1016/S0022-2828(08)80035-1 7760360

[B39] Kruger AL Peterson S Turkseven S Kaminski PM Zhang FF Quan S Wolin MS Abraham NG D-4F induces heme oxygenase-1 and extracellular superoxide dismutase, decreases endothelial cell sloughing, and improves vascular reactivity in rat model of diabetes Circulation 2005 111 3126 3134 10.1161/CIRCULATIONAHA.104.517102 15939814

[B40] Dunlay SM Weston SA Redfield MM Killian JM Roger VL Tumor necrosis factor-alpha and mortality in heart failure: a community study Circulation 2008 118 625 631 10.1161/CIRCULATIONAHA.107.759191 18645056 PMC2682349

[B41] Satoh M Minami Y Takahashi Y Nakamura M Immune modulation: role of the inflammatory cytokine cascade in the failing human heart Curr Heart Fail Rep 2008 5 69 74 10.1007/s11897-008-0012-2 18765076

[B42] Li Volti G Seta F Schwartzman ML Nasjletti A Abraham NG Heme oxygenase attenuates angiotensin II-mediated increase in cyclooxygenase-2 activity in human femoral endothelial cells Hypertension 2003 41 715 719 10.1161/01.HYP.0000049163.23426.66 12623985

[B43] Kwak JY Takeshige K Cheung BS Minakami S Bilirubin inhibits the activation of superoxide-producing NADPH oxidase in a neutrophil cell-free system Biochim Biophys Acta 1991 1076 369 373 10.1016/0167-4838(91)90478-I 1848104

[B44] Turkseven S Kruger A Mingone CJ Kaminski P Inaba M Rodella LF Ikehara S Wolin MS Abraham NG Antioxidant mechanism of heme oxygenase-1 involves an increase in superoxide dismutase and catalase in experimental diabetes Am J Physiol Heart Circ Physiol 2005 289 H701 H707 10.1152/ajpheart.00024.2005 15821039

[B45] Erdei N Toth A Pasztor ET Papp Z Edes I Koller A Bagi Z High-fat diet-induced reduction in nitric oxide-dependent arteriolar dilation in rats: role of xanthine oxidase-derived superoxide anion Am J Physiol Heart Circ Physiol 2006 291 H2107 H2115 10.1152/ajpheart.00389.2006 16798827

[B46] Roberts CK Barnard RJ Sindhu RK Jurczak M Ehdaie A Vaziri ND Oxidative stress and dysregulation of NAD(P)H oxidase and antioxidant enzymes in diet-induced metabolic syndrome Metabolism 2006 55 928 934 10.1016/j.metabol.2006.02.022 16784966

[B47] Galili O Versari D Sattler KJ Olson ML Mannheim D McConnell JP Chade AR Lerman LO Lerman A Early experimental obesity is associated with coronary endothelial dysfunction and oxidative stress Am J Physiol Heart Circ Physiol 2007 292 H904 H911 17012356 10.1152/ajpheart.00628.2006

[B48] Mathew AV Okada S Sharma K Obesity related kidney disease Curr Diabetes Rev 2011 7 41 49 10.2174/157339911794273928 21067508

[B49] Ix JH Sharma K Mechanisms linking obesity, chronic kidney disease, and fatty liver disease: the roles of fetuin-A, adiponectin, and AMPK J Am Soc Nephrol 2010 21 406 412 10.1681/ASN.2009080820 20150538 PMC4473254

[B50] Ran J Xiong X Liu W Guo S Li Q Zhang R Lao G Increased plasma adiponectin closely associates with vascular endothelial dysfunction in type 2 diabetic patients with diabetic nephropathy Diabetes Res Clin Pract 2010 88 177 183 10.1016/j.diabres.2010.01.021 20138682

[B51] Sambuceti G Morbelli S Vanella L Kusmic C Marini C Massollo M Augeri C Corselli M Ghersi C Chiavarina B Rodella LF L'Abbate A Drummond G Abraham NG Frassoni F Diabetes Impairs the Vascular Recruitment of Normal Stem Cells by Oxidant Damage; Reversed by Increases in pAMPK, Heme Oxygenase-1 and Adiponectin Stem Cells 2009 27 399 407 10.1634/stemcells.2008-0800 19038792 PMC2729677

[B52] Merrill GF Kurth EJ Hardie DG Winder WW AICA riboside increases AMP-activated protein kinase, fatty acid oxidation, and glucose uptake in rat muscle Am J Physiol 1997 273 E1107 E1112 9435525 10.1152/ajpendo.1997.273.6.E1107

[B53] Kudo N Gillespie JG Kung L Witters LA Schulz R Clanachan AS Lopaschuk GD Characterization of 5'AMP-activated protein kinase activity in the heart and its role in inhibiting acetyl-CoA carboxylase during reperfusion following ischemia Biochim Biophys Acta 1996 1301 67 75 8652652 10.1016/0005-2760(96)00013-6

[B54] Terai K Hiramoto Y Masaki M Sugiyama S Kuroda T Hori M Kawase I Hirota H AMP-activated protein kinase protects cardiomyocytes against hypoxic injury through attenuation of endoplasmic reticulum stress Mol Cell Biol 2005 25 9554 9575 10.1128/MCB.25.21.9554-9575.2005 16227605 PMC1265833

[B55] Schulz E Dopheide J Schuhmacher S Thomas SR Chen K Daiber A Wenzel P Munzel T Keaney JF Jr Suppression of the JNK pathway by induction of a metabolic stress response prevents vascular injury and dysfunction Circulation 2008 118 1347 1357 18809807 10.1161/CIRCULATIONAHA.108.784298 PMC2756193

[B56] Dimmeler S Fleming I Fisslthaler B Hermann C Busse R Zeiher AM Activation of nitric oxide synthase in endothelial cells by Akt-dependent phosphorylation Nature 1999 399 601 605 10.1038/21224 10376603

[B57] Chen ZP Mitchelhill KI Michell BJ Stapleton D Rodriguez-Crespo I Witters LA Power DA Ortiz De Montellano PR Kemp BE AMP-activated protein kinase phosphorylation of endothelial NO synthase FEBS Lett 1999 443 285 289 10.1016/S0014-5793(98)01705-0 10025949

